# Productivity of Spitsbergen fjords ecosystems in summer—Spatial changes of in situ primary production in Kongsfjorden and Hornsund in the period 1994–2019

**DOI:** 10.1002/ece3.11607

**Published:** 2024-06-25

**Authors:** Katarzyna Dragańska‐Deja, Joanna Stoń‐Egiert, Józef Wiktor, Mirosława Ostrowska

**Affiliations:** ^1^ Remote Sensing Laboratory, Department of Marine Physics, Institute of Oceanology Polish Academy of Sciences Sopot Poland; ^2^ Marine Bio‐Optic Laboratory, Department of Marine Physics, Institute of Oceanology Polish Academy of Sciences Sopot Poland; ^3^ Marine Protists Laboratory, Department of Marine Ecology, Institute of Oceanology Polish Academy of Sciences Sopot Poland

**Keywords:** chlorophyll a, ^14^C isotope method, Hornsund, Kongsfiord, primary production, Spitsbergen fjords

## Abstract

This comprehensive study examines primary production (PP) within the Spitsbergen fjords, Hornsund, and Kongsfjord, over a 25‐year period (1994–2019), across 45 stations and 348 incubation levels at various depths. PP and hydrological parameters were measured at 28 sampling stations in Kongsfjorden and 17 in Hornsund, with the locations of “Glacier,” “Inner,” and “Outer” zones defined to reflect the varying influence of glacial meltwater. Our study revealed spatial and temporal variability in PP, both at the surface and within the water column with very high depth resolution. The highest PP values were observed in the Glacier and Inner zones of Hornsund, particularly in the water layer up to 3 m depth, exceeding 20 mgC m^−3^ h^−1^. A notable decline in PP with increasing depth was observed in both fjords, with the Glacier zones displaying the highest productivity at the surface. The study also highlights the influence of glacial meltwater on surface water conditions, affecting the PP in the upper layers of both fjords. The observed gradient in the depth of maximum PP toward the mouth of the fjord varied between the two fjords, with Kongsjord displaying more dynamic variations. The spatial distribution of integrated primary production (Pi) suggested lower productivity in the glacial regions, likely due to light limitation caused by high concentrations of mineral particulate matter. The values of Pi were considerably higher in Hornsund, approximately twice as high overall, with specific emphasis on the Glacier and Inner zones where Pi values were about 6.5 and 2.5 times higher, respectively, when compared to those observed in Kongsfjord.

## Graphical abstract

This comprehensive study examines primary production (PP) within the Spitsbergen fjords, in “Glacier,” “Inner,” and “Outer” zones of Hornsund and Kongsfjord, over a 25‐year period (1994–2019), involving 45 experiments and 348 incubations levels at various depths. Our study revealed significant spatial and temporal variability in PP, both at the surface and within the water column and also highlighted the influence of glacial meltwater affecting the PP in the upper layers of both fjords.

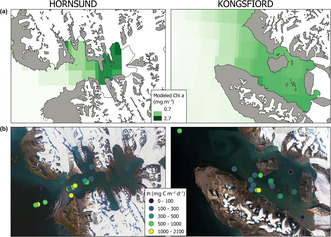

## 1 INTRODUCTION

The climate changes taking place on Earth have substantially modified the physical, chemical, and biological properties of the ocean, with pronounced effects in the polar regions. The Arctic ecosystem is highly sensitive to climate warming, as manifested not only in the reduction of summer sea ice cover and volume by about 50% in the last 40 years (Serreze & Meier, 2019) but also in changing thermal conditions, ocean acidification, changing nutrient concentrations and including shifts in the biodiversity of phytoplankton community structure (Gao et al., 2019; Rousseaux & Gregg, 2015; Tate et al., 2017). Within coastal zones, the effect of melting glaciers also lowers the transparency of water, which in turn affects solar‐stimulated processes like photosynthesis. During this process, marine autotrophs convert dissolved inorganic carbon into organic matter, thereby playing a pivotal role in oceanic ecosystems. As a result, primary production (PP) is a fundamental ecosystem service supplying energy to the entire marine food web. This process is governed by light and nutrient availability, phytoplankton grazing (Hopwood et al., 2020), and water column stratification. PP is the basis of the food chain fueling the ecosystem with energy, and playing a pivotal role in the global carbon cycle and atmospheric oxygen supply. Knowledge of this phenomenon is very important for understanding the functioning of Earth's ecosystems, particularly as it is estimated that nearly half of all organic carbon is produced in the oceans (Field et al., 1998). Understanding and predicting changes in ocean productivity necessitate consideration of both global/pan‐Arctic and local (glacier‐fjord) scales. Global climate drivers set the context, but localized studies reveal the specific ecological responses and nutrient dynamics unique to glacier‐influenced fjords. Each scale presents distinct processes and effects, which are essential for comprehensive insights into ocean productivity. This is evident in the Polar regions, where the effects of these changes are more pronounced than in midlatitudes, as demonstrated by studies such as Overland et al. (2019) and Rinke and Dethloff (2008) as well as the 2018 IPCC report. Studies have shown that in regions of a Greenland fjord most affected by meltwater, the rate of bacterial degradation surpasses that of new production by phytoplankton, resulting in a net heterotrophic ecosystem. As the distance from the meltwater source increases, a gradual transition to an autotrophic system occurs in the outer fjord, where production exceeds degradation (Sejr et al., 2022).

Estimates of ocean primary productivity based on satellite observations in the Northern Hemisphere exhibit positive trends over 2003–2021. In the nine regions investigated across the Arctic (Eurasian Arctic, Amerasian Arctic, Sea of Okhotsk, Bering Sea, Barents Sea, Greenland Sea, Hudson Bay, Labrador Bay, and North Atlantic), the strongest trends occurred in the Eurasian Arctic and Barents Sea, indicated from 7% to as much as 59% change over this period (Frey et al., 2021).

Monitoring changes in the marine environment is a critical issue in contemporary oceanology and necessitates the use of precise measurement techniques. Satellite observations, which have become a prevalent source of data, offer systematic information from extensive areas. The use of satellite data has revolutionized our ability to study the Arctic (Comiso & Hall, 2014; Konik et al., 2020, 2021; Urbanski et al., 2017). Therefore, most of the analyses presented in the literature are based on data received from bio‐hydrological models, which use satellite‐derived data. These studies cover vast areas (e.g., the North Atlantic, the Canadian or Eurasian Arctic, and the Barents Sea) and deal mainly with the general flows of water masses in the Arctic region. The undoubted advantage of these computed estimates is the year‐round prediction of marine ecosystem parameters such as temperature or chlorophyll *a* concentration (Garnesson et al., 2019; Uitz et al., 2010). Despite the fact that remote sensing data provide a consistent, and comprehensive record suitable for parameter‐change, trend studies need to be validated with in situ measurements (Comiso & Hall, 2014; Minnett et al., 2019). Satellite data provide reliable information only about the subsurface layer of open water. To investigate changes in biogeochemical parameters within the entire water column, especially in coastal ecosystems, it is imperative to conduct in situ measurements to verify and calibrate commonly used models (Blondeau‐Patissier et al., 2014; Oziel et al., 2022).

There is little information on measurements of PP in situ in the polar regions. There are several methods (e.g., oxygen production and ^14^C assimilation) to carry out such measurements quasi directly in the environment. However, these methods are laborious and require specialized equipment, as well as conducting measurements on research vessels. Models that utilize the knowledge of surface temperature, surface concentration of chlorophyll *a* and photosynthetically available radiation (PAR) on the surface layer are often used to estimate the amount of organic matter produced by algae (Ficek et al., 2003; Woźniak et al., 2003), but their accuracy requires continuous validation against in situ measurements, especially in areas with dynamic environmental changes.

The west coast of Spitsbergen serves as a natural laboratory for studying changes, particularly in the interaction between Atlantic and Arctic water masses and their consequences, such as phytoplankton composition and PP, which are visibly evident (Assmy et al., 2023; Hegseth et al., 2019; Wiktor et al., 2022; Wiktor & Wojciechowska, 2005). These changes are particularly visible in the fjords (Kongsfjord and Hornsund), where the biogeochemical conditions of the waters are additionally affected by the waters from the melting glaciers. Both fjords are under the influence of relatively warm Atlantic‐derived water (with summer temperatures around 4–6°C on the western coast), although Hornsund is more influenced by cold water (<0°C) from the eastern coast (Piwosz et al., 2009; Promińska et al., 2017; Svendsen et al., 2002). Additionally, the runoff of freshwater from the glaciers with large amounts of mineral particles reduces not only the thickness of the euphotic layer and changes light propagation conditions (Konik et al., 2020; Svendsen et al., 2002) but also creates very strong salinity stratification of the water column (Promińska et al., 2018; Svendsen et al., 2002). Surface water salinity can be reduced to less than 28 in the inner part of Kongsfjorden. Błaszczyk et al. (2019) reported that up to an equivalent of 9% of the fjord volume of Hornsund may originate from glacier freshwater runoff every year. Such changes in environmental conditions cause significant differences in the composition and the quantitative ratios of plankton biota, with the most dramatic variation in the number of microplankton taxa and shift into nanoplanktonic ones in summer and their biomass (Wiktor et al., 2022), and thus significantly affect the efficiency of organic matter production processes. Diatoms were the most substantial contributors to the organic part of suspended matter, especially during spring (Assmy et al., 2023; Wiktor, 1999) in the outer basins of both fjords, whereas the second most important contributors were auto‐ and heterotrophic dinofagellates, and nanoflagellates in Kongsfjorden (Hegseth et al., 2019; Hop et al., 2002) and nanofagellates in Hornsund (Piwosz et al., 2009). In early spring (March–April), the return of sunlight initiates PP, with abundant nutrients due to winter mixing. In spring (May–June), increased light and stable stratification lead to rapid phytoplankton growth, dominated by diatoms (Hodal et al., 2012). As the bloom progresses, surface nutrients become depleted. During summer (July–August), the post‐bloom phase sees a decrease in phytoplankton biomass, with a shift to smaller phytoplankton like flagellates and picoplankton. Episodic mixing events can increase nutrient levels, causing smaller secondary blooms (Piwosz et al., 2009). In autumn (September–October), decreasing daylight reduces PP, and the water column mixes, redistributing nutrients and transitioning to a winter state with minimal biological activity (Assmy et al., 2023).

Documented changes in the content of indicator pigments for these groups, analyzed in sediments, indicate their variable biomass occurring in both fjords in different years (Krajewska et al., 2020).

There are only a few in situ measurements of PP from the polar region available in the literature, and they are limited to the spring–summer season due to the light cycle and accessibility to the area (Hodal et al., 2012; Hop et al., 2002; Iversen & Seuthe, 2011; Smoła et al., 2017; Van De Poll et al., 2018). Late Autumn and Winter are considered non‐productive seasons and only one publication assesses PP in December in Kongsfjorden (Iversen & Seuthe, 2011).

The aim of this study is to analyze the in situ measured values of PP in two Spitsbergen fjords, Kongsfjorden and Hornsund, during a 25‐year period (1994–2019) to determine the variability resulting from the influence of melting glacier waters and Atlantic waters (AW) on the amount of organic matter produced in the summer season. Characterization of PP in the water column, at the surface layer and values integrated into the water column was possible to conduct on the base of 45 measurement experiments in different parts of the fjords. Furthermore, the potential use of satellite data for assessing chlorophyll *a* concentration in fjord environments was explored. A comparative analysis was conducted between the values obtained from satellite‐based chlorophyll *a* products and direct measurements taken in the field. The results presented in this study are a unique and comprehensive set of environmental data that document the magnitude and direction of changes in dynamically changing Arctic fjord ecosystems.

## 2 MATERIALS AND METHODS

### 2.1 Sampling design and study area

**FIGURE 1:**
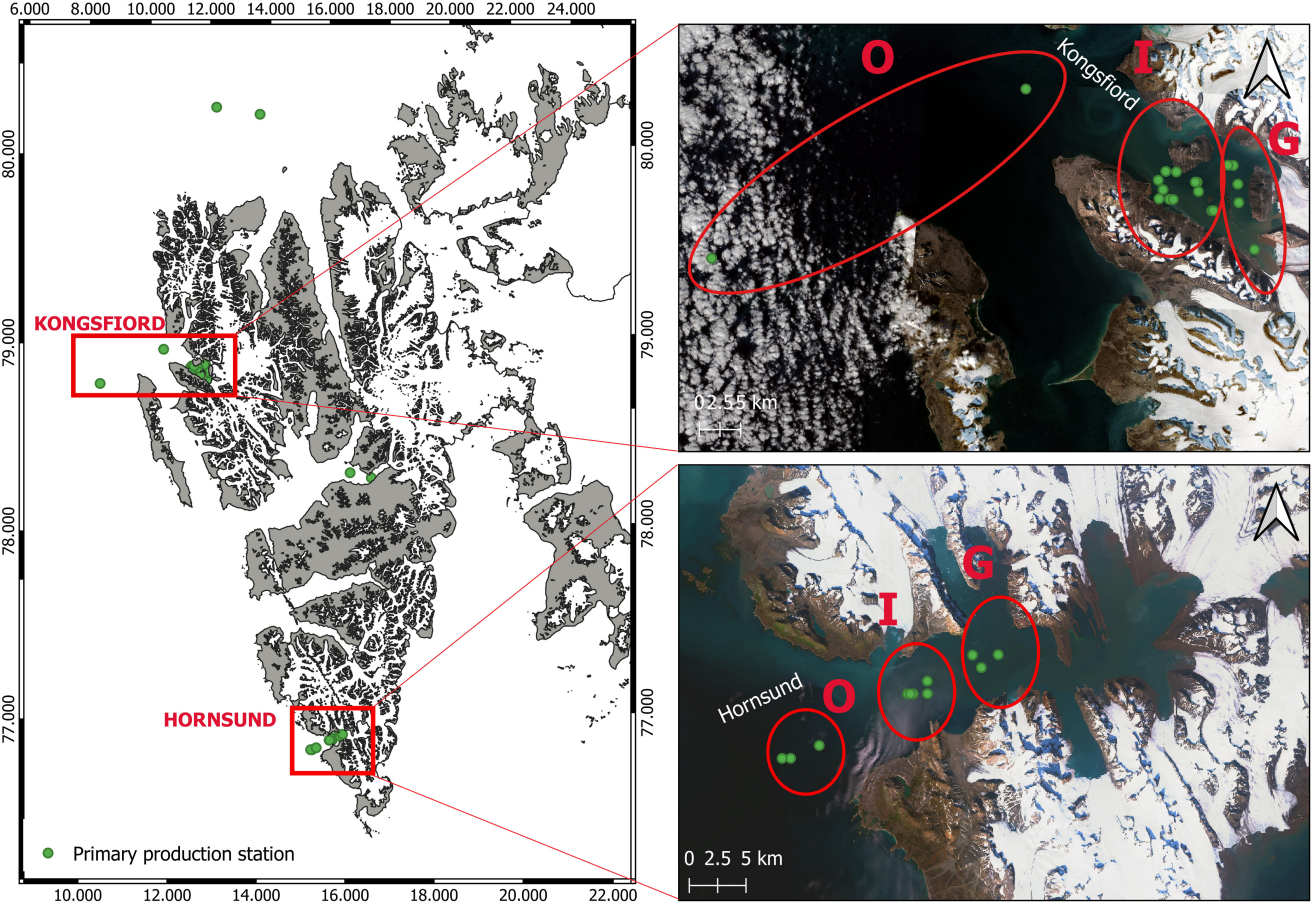
Location of Kongsfjorden and Hornsund fjord on the Svalbard Archipelago and the location of the measuring stations in the Outer (O), Inner (I), and Glacier (G) zones of both fjords.

**FIGURE 2:**
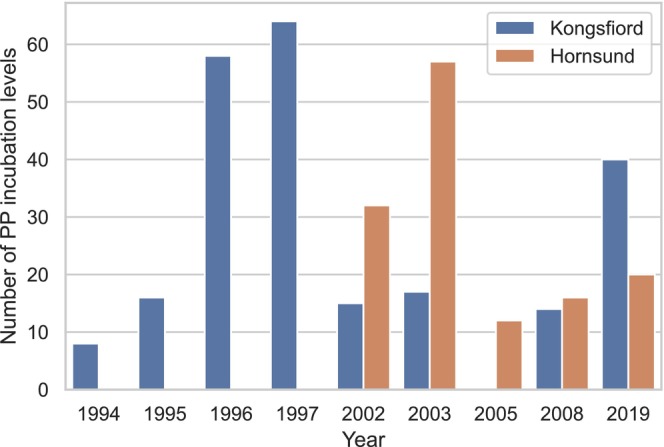
The number of primary production incubations conducted in Kongsfjorden and Hornsund fjord within the period 1994–2019.

In total, 45 expositions of PP were conducted during the summer months (July–August) in the years 1994–2019 at Spitsbergen fjords: Hornsund and Kongsfjord (Figure 1). Altogether, a total of 348 incubations were performed at different depths to estimate PP, which included 137 incubation levels conducted at Horsund and 232 levels, carried out at Konsfjord (Figure 2). These two glaciated fjords are on opposite ends location: Kongsfjord is located at 79° N and Hornsund is located southward (77° N). From a hydrological regime perspective, Kongsfjorden is under the stronger influence of relatively warm Atlantic‐derived water (Cottier et al., 2010; Hegseth et al., 2019), while Hornsund receives a temporary flowing the cold Arctic water from the Barents Sea (Promińska et al., 2017).

Measurements of PP in the water column and additional hydrological parameters were carried out on board RV *Oceania* at 28 measurement stations in Kongsfjorden and 17 in Hornsund (Figure 1). Due to the different inflow of meltwater from glaciers and thus different environmental conditions, both fjords were divided into three subregions: Glacier (G), Inner (I), and Outer (O) zone, taking into account the decreasing impact from the glacier melt surface waters.

### 2.2 Methods of measurements

#### 2.2.1 Primary production

The PP measurements were carried out with ^14^C light and dark bottles according to Nielsen (1952), Nielsen and Bresta (1984), and Strickland and Parsons (1972). The idea of this method is to calculate the rate of the PP in distinct samples on the basis of the known total content of CO_2_ in water and the incorporation of the tracer into an organic matter of phytoplankton cells during photosynthesis.

Water samples were taken at 10 distinct depths in layers 0–50 m by Niskin bottle. This selection aimed to capture the variation in vertical distribution of algal biomass, and specific hydrological and light conditions at the sampling stations. Subsamples of 100 mL water were inoculated with labeled sodium bicarbonate NaH^14^CO_3_ to obtain radioactivity of 8 μCi per subsample and incubated in transparent glass bottles at the depths of collection. Expositions were mainly carried out during the day between 06:00 and 18:00 UTC. To avoid the potential shading, the samples were attached to a drifting buoy about 50 m from the ship. After the incubation in natural environmental conditions, samples were filtered through a cellulose acetate filter of 25 mm diameter and pore size of 0.45 μm (Sartorius brand). The filters were exposed to HCl acid fumes for 5 min, dried for 24 h, and placed in scintillation vials for further analysis of radioactivity.

At the laboratory, filters were dissolved in scintillation liquid and radioactivity was analyzed with a scintillation counter (Beckman LS 6000 IC).

An amount of carbon fixed into the new organic matter per subsample during exposition at depth *z* [Pe(*z*)], was calculated according to the following equation (Nielsen & Bresta, 1984), where organic ^14^C losses from phytoplankton respiration and the effect of ^14^C discrimination were compensated by appropriate factors:

(1) $$\mathrm{Pe}\left(z\right)=\frac{<{\mathrm{dpm}}_{a}\left(z\right)>∙\text{total}\;CO_{2}∙13.356∙k_{1}∙k_{2}∙k_{3}}{{\mathrm{dpm}}_{b}}\left[\mathrm{mg}\;C\;m-3\;h-1\right]$$

where <dpma(*z*)> —the mean of differences between the activities of light and dark bottles at depth *z* [dpm]; dpmb—the activity of isotope ^14^C [dpm]; total CO_2_—the total carbon concentration in the water sample [mM dm^−3^]; 13.356—the results from the multiplication of the following parameters: 12—the atomic weight of carbon; 1.05—a correction of the effect of ^14^C; the uptake of the ^14^C isotope is 5% slower than that of the ^12^C isotope found in nature; 1.06—a correction of the respiration of organic matter produced during the experiment, equals 6% at optimal photosynthesis; *k_1_*—a correction of subsampling factor; *k_2_*—a time factor, for example, used to convert production per 2 h to production per hour, then *k_2_* = 0.5; and k_3_—a dimension factor, for example, used to convert mgC dm^−3^ to mgC m^−3^, *k_3_* = 10^3^.

The inorganic carbon concentration was calculated according to an algorithm given by Strickland and Parsons (1972) with measured in situ temperature, salinity, and pH of water samples. The temperature (T) and salinity (S) profiles were conducted using a conductivity–temperature–depth probe.

The values of integrated PP under a square meter of sea surface area of the investigated water column, signed as Pi (mg C m^−2^ day^−1^) were calculated using the numerical integration at discrete depths in the water column:

(2) $$\mathrm{Pi}\left(0-30\right)=\int_{z=0}^{30}\mathrm{Pe}\left(z\right)\mathrm{dz}$$

#### 2.2.2 Chlorophyll *a* concentration

The subsamples for the determination of chlorophyll *a* concentration at the 10 sampled depths were taken at the same time as PP samples and filtered immediately under the low vacuum conditions through 47 mm Whatman GF/F filters with a 0.7 μm nominal particle retention. The filters were frozen immediately after filtration in −80°C or in liquid nitrogen. In the laboratory, chlorophyll *a* was extracted in 96% ethanol for 24 h in darkness and absorbance measurements of extracts were performed by spectrophotometers UV4‐100 Unicam and Perkin Elmer Lambda 650. The concentration of chlorophyll *a* was calculated according to Strickland and Parsons (1972).

#### 2.2.3 Chlorophyll *a* satellite data

The chlorophyll *a* satellite data derived from MODIS/AQUA were obtained from the OceanColor website (National Aeronautics and Space Administration, NASA, https://oceancolor.gsfc.nasa.gov/l3/) archive of Level‐3 gridded data of 4 km. To obtain the average concentration of chlorophyll *a* in July, Monthly Climatology data were used, which are a composite of all data collected during a single month across 2002–2022.

Maps and spatial analyses were made using QGIS 3.16.3 software. All plots and statistical analyses were conducted using Python 3.7 using Matplotlib 3.1.1, Pandas 1.0.5, and Seaborn 0.12.0.

To determine whether the regions differ statistically, we conducted a one‐way analysis of variance (ANOVA) on the data, having first validated the assumptions of normality and homogeneity of variances using the Shapiro–Wilk test and Levene's test, respectively. For the non‐normal data specific to Konfsfjoden, we employed the Kruskal–Wallis test as a non‐parametric alternative to ANOVA.

## 3 RESULTS

### 3.1 Environmental conditions

#### 3.1.1 Hydrology

**FIGURE 3:**
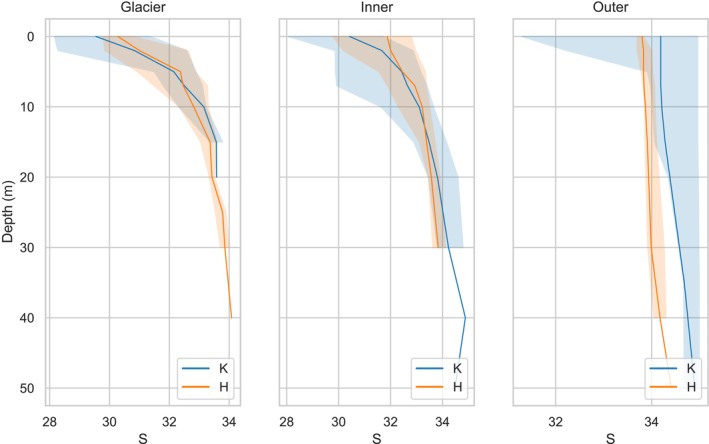
Vertical profiles of median value (solid line) of salinity S with min–max values (shading) in Glacier, Inner, and Outer parts of Kongsfjord (K) and Hornsund (H) fjord.

**FIGURE 4:**
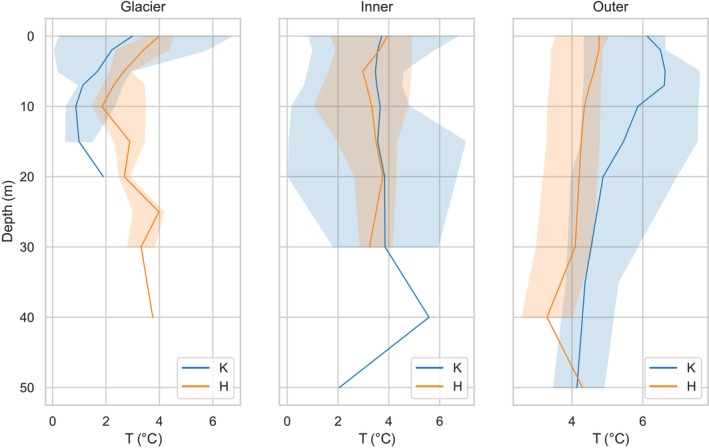
Vertical profiles of median value (solid line) of temperature (T) with min–max values (shading) in Glacier, Inner, and Outer parts of Kongsfjord (K) and Hornsund (H) fjord.

Due to the variation in water salinity and temperature resulting from the melting of glaciers, fjords have been divided into three subregions: the Glacier (G), Inner (I), and Outer (O) zones (Figures 3 and 4). These zones were established based on the diminishing influence of glacier melt surface waters on the surrounding environment as the distance from the glacier increases. Based on the empirical data gathered within the Glacier zones, it has been observed that the average surface salinity (0 m) during the study period at Hornsund was 30.28 ± 0.52, while at Kongsfjord, it was found to be slightly lower, at 29.65 ± 1.36. Concurrently, the average temperatures in the entire water column in these zones were measured at 3.08 ± 0.79°C for Hornsund and 2.15 ± 1.80°C for Kongsfjord, respectively. This notable variance in temperatures between the two localities can be ascribed to the proximity of the measurement points in Kongsfjord to marine‐terminating glaciers. The furthest sampling point from the glacier front is approximately 6 km away in Kongsfjord and around 9 km away (to Paielrbreen in Burgerbukta) in Hornsund. Such proximity led to a more pronounced influence of the colder meltwater in this region. Furthermore, in the Inner zones of both fjords, the average surface salinity and temperature were closely matched, with Hornsund recording 31.61 ± 0.87°C and 3.61 ± 1.13°C, and Kongsfjord exhibiting values of 30.40 ± 1.28°C and 3.77 ± 1.27°C. Notably, the Outer zones of both fjords exhibited a significant increase in surface salinity levels, with Hornsund showing a salinity of 33.78 ± 0.06 and Kongsfjord reaching 33.48 ± 1.58. Correspondingly, the average surface temperatures in these Outer zones were 4.46 ± 0.64°C for Hornsund and a markedly higher 5.69 ± 0.97°C for Kongsfjord. This disparity in temperature is indicative of the influence of warm AW in Kongsfjord, in contrast to the predominantly colder waters prevailing in Hornsund.

**FIGURE 5:**
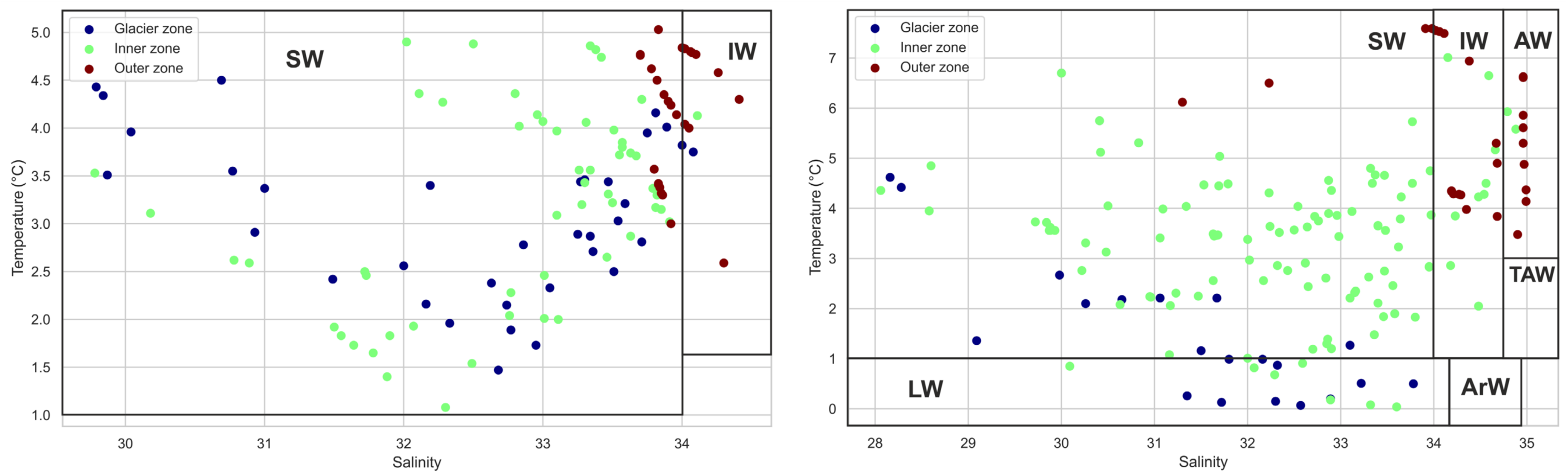
T–S diagram of the water masses that have been identified in Hornsund (left panel) and Kongsfjord (right panel) during field campaigns. ArW, Arctic Water; AW, Atlantic Water; IW, Intermediate Water; LW, Local Water; SW, surface water; TAW, Transformed Atlantic Water.

The T–S diagram was used to classify water masses using measured temperature and salinity data according classification system proposed by Cottier et al. (2005) and Promińska et al. (2017) (Figure 5). The classification indicates that in the summer, surface water (SW), which is characterized by salinity levels below 34.00 and temperatures greater than 1°C, is the most prevalent type of water in the Spitsbergen fjord. This water is formed under glacier meltwater influence (Svendsen et al., 2002). In all three groups of investigated zones, SW waters were detected. In terms of water masses, the Inner region shares significant similarities with the Glacier region. However, it is worth noting that the occurrence of intermediate water (IW) was observed at deeper layers in the Inner region, whereas in the Glacier region, SW and Local Water masses were present throughout the entire water column. The Outer region is different and easily distinguishable due to the presence of IW in Hornsund and an additional AW in Kongsfjord (Figure 5).

#### 3.1.2 Chlorophyll *a* concentration in the water column

**FIGURE 6:**
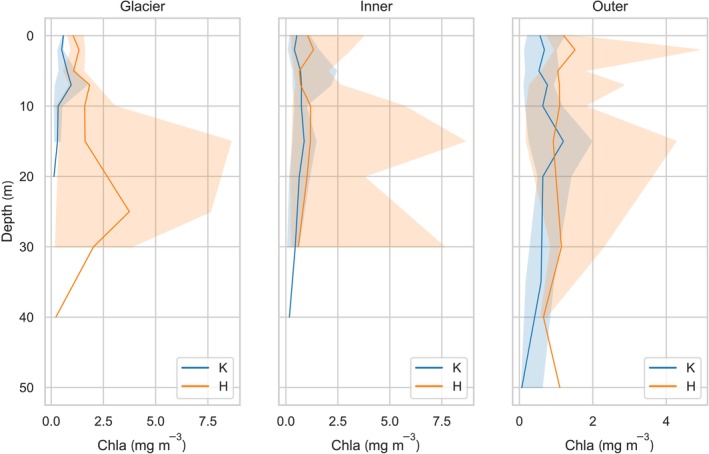
Vertical profiles of the median value of chlorophyll *a* concentration Chla (mg m^−3^) with min–max values in the investigated areas.

The variability in the concentrations of chlorophyll *a* within the water column across various sectors of both fjords is delineated in Figure 6. Chlorophyll *a*, serving as a crucial pigment in photosynthesis and also an indicator of the autotrophs fraction of suspended matter, exhibits a wide range of concentration levels. This variance is indicative of substantial fluctuations in the phytoplankton biomass within the period under examination. Notably, Arctic phytoplankton demonstrates distinct zonation within the water column. This zonation is reflective of the presence of areas with markedly more conducive conditions for the cell development of algal organisms from diverse classes (Hegseth et al., 2019; Sukhanova et al., 2009).

The highest concentrations of chlorophyll *a* (8.61 mg m^−3^), were detected in Hornsund, particularly in the Glacier and Inner zones at approximately 15 m. The low chlorophyll *a* concentrations in the surface water of the Glacier zone suggest the dominance of a layer of glacial meltwater, where the organic particulate matter is minimal. On the other hand, the vertical profiles of chlorophyll *a* in Kongsfjord exhibit less variation depending on the specific zone. It is observed that as the distance from the glacier increases, the maximum of the median distribution in chlorophyll *a* concentrations gradually shifts to deeper layers, ranging from 7 m in the Glacier zone to 15 m in the Outer zone of the fjord.

### 3.2 Changes in primary production

#### 3.2.1 Primary production in the water column

**FIGURE 7:**
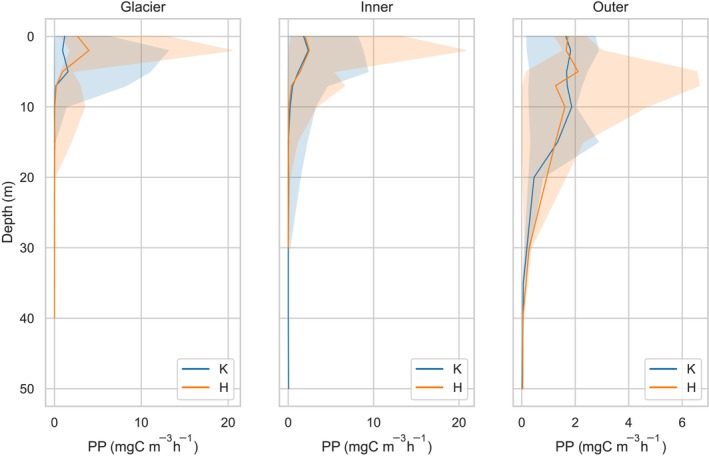
Vertical profiles of the median value of primary production—Pe (mg C m^−3^ h^−1^) with min–max values in Glacier, Inner, and Outer parts of Kongsfjord and Hornsund fjord.

The data obtained on the vertical distribution of PP are presented in Figure 7, which illustrates the variations depending on the specific zones analyzed in both Hornsund and Kongsfjord locations. The highest values of PP were recorded in 2008 in the Glacier and Inner zones of Hornsund fjord at a depth of 3 m and its value exceeded 20 mgC m^−3^ h^−1^ (Figure 7). In the Glacier zone, the highest PP values were observed in the water layer up to a depth of 3 m. As the depth increased to 7 m, the PP values decreased significantly, with the value at this depth ranging from 0.19 to 0.52 mgC m^−3^ h^−1^. Furthermore, at a depth of 10 m, the PP values were negligible, with the value at this depth no longer exceeding 0.05 mgC m^−3^ h^−1^. In the Glacier zone at Kongsfjord, the maximum value for PP was also recorded in the surface layer, although the maximum value did not exceed 13.1 mgC m^−3^ h^−1^. Unlike at Hornsund, the PP did not decrease as significantly with depth. At a depth of 10 m, the value was up to 1.36 mgC m^−3^ h^−1^, and at 15 m, it dropped to 0.07 mgC m^−3^ h^−1^.

The Inner zone is significantly influenced by surface water originating from melting glaciers. This zone also displays the highest levels of PP in the top 3 m, which range from 0.01 mgC m^−3^ h^−1^ at 0 m to 8.75 mgC m^−3^ h^−1^ at 3 m in Kongsfjord and from 0.11 mgC m^−3^ h^−1^ to 20.75 mgC m^−3^ h^−1^ in Hornsund. Although the average production values at depths of 10–15 m were relatively low, single measurements even at 20 m recorded values of 1.45 mgC m^−3^ h^−1^ for Hornsund and 1.4 mgC m^−3^ h^−1^ for Kongsfjord. The values of PP exceeding 1 mgC m^−3^ h^−1^ were found to be deeper in the Inner area than in the Glacier zone.

In the Outer zone, the highest PP values, exceeding 6.5 mgC m^−3^ h^−1^, were documented within the 5–7 m depth layer. In contrast, the Kongsfjord exhibited a distinct pattern with two notable maxima in primary production. The maximum value in this fjord was considerably lower, reaching only 2.86 mgC m^−3^ h^−1^, and was observed at two distinct depths: 2 and 15 m. This variation in primary production values and their distribution across different depths highlights the spatial heterogeneity in the ecological dynamics of these fjord systems and points to the complex interplay of environmental factors influencing primary production at varying depths.

**FIGURE 8:**
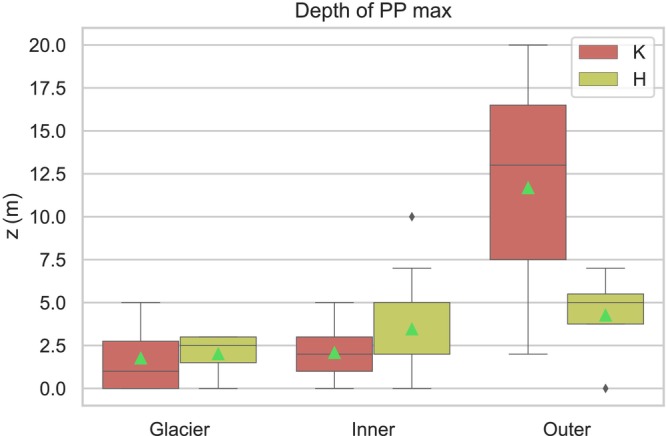
Boxplots illustrating the depth distribution of the maximum primary production [Pe (mg C m^−3^ h^−1^)] value within the water column across the Glacier, Inner, and Outer zones in Hornsund (H) and Kongsfjord (K). The boxplot delineates the quartiles of the dataset, with the central box representing the interquartile range and the “whiskers” extending to depict the remainder of the data distribution, excluding points identified as outliers. The mean value of each dataset is denoted by a green triangle.

The observed depth of maximum PP within the fjords exhibits a notable variation, increasing toward the mouth of the fjord, with Kongsfjord displaying particularly dynamic variations, as illustrated in Figure 8. In Kongsfjord, there is a marked variation in the average depth at which the maximum of PP is observed, ranging from a shallow 2 m in the Glacier zone to a significantly deeper 15 m in the Outer zone. In contrast, Hornsund presents a more constrained range of depth for maximum PP, spanning from 2 m near the glacier front to a moderately deeper range of 5–7 m in the Outer zone of the fjord. This observed pattern not only highlights the spatial variability within each fjord in terms of the depth at which maximum PP occurs but also underscores the distinct differences between Kongsfjord and Hornsund. These differences are particularly evident in the varied water masses observed in the Outer zones of both fjords.

#### 3.2.2 Surface primary production

**FIGURE 9:**
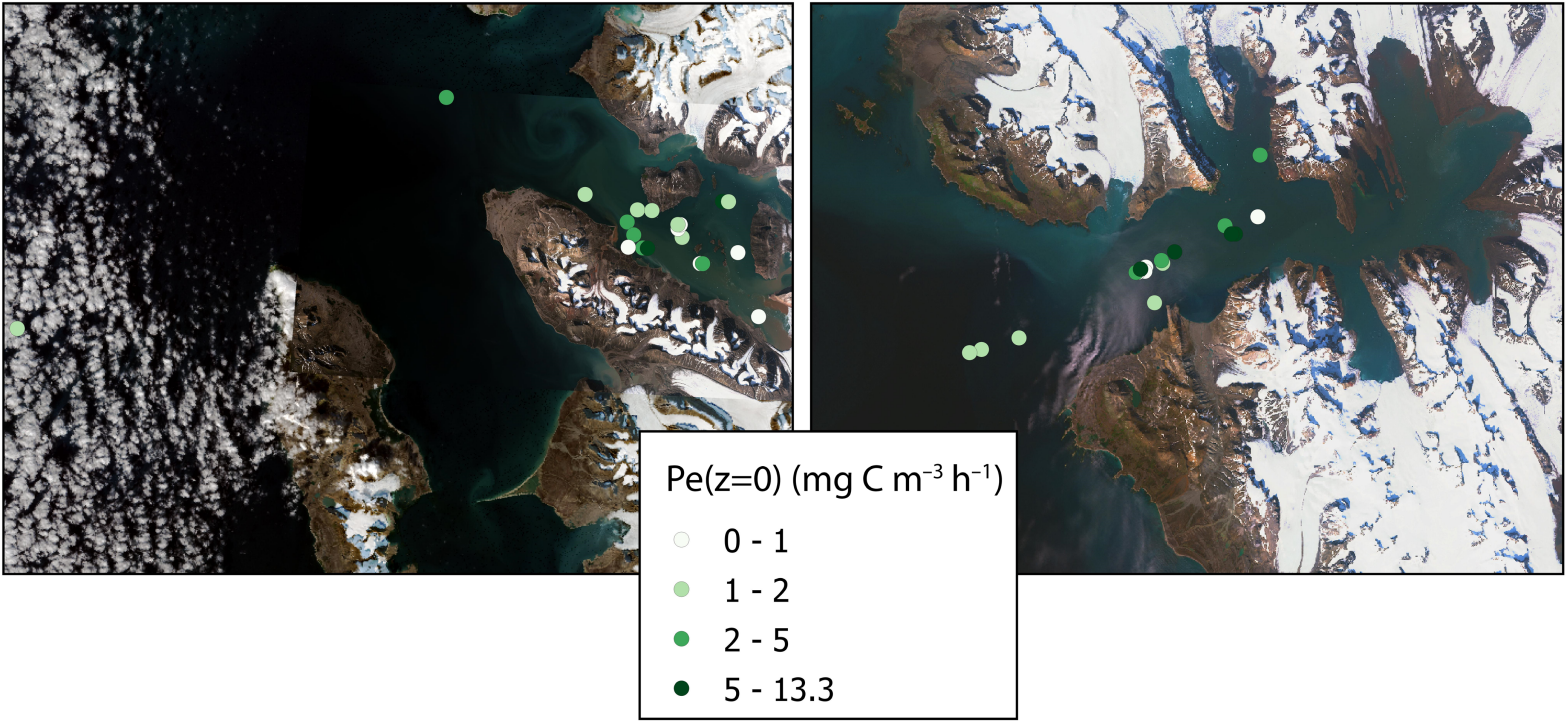
Spatial variability of surface—Pe(0) (mg C m^−3^ h^−1^) values of primary production in Kongfjord and Hornsund.

Variations in surface values of primary production Pe(0), temperature T(0), salinity S(0), and chlorophyll *a* concentration Ca(0) in the Kongsfjorden and Hornsund fjord are detailed in Table 1 and illustrated in Figure 9. Despite the similarity in average temperatures in the inner parts of both fjords (Glacier and Inner zones), the measured parameters in Kongsfjord consistently registered lower values compared to those in Hornsund fjord. Notably, Pe(0) values in the Glacier part of Hornsund were 2.8 times higher, and 1.6 times higher in the Inner part, relative to the average values measured in Kongsfjord. The least disparity was observed in the Outer parts of the fjords, where values were comparable, with Hornsund exhibiting only a 1.2‐fold increase over Kongsfjord.

**TABLE 1:** Ranges (min–max), mean, and standard deviation of the surface values of primary production—Pe(0) (mg C m^−3^ h^−1^), temperature—T(0) (°C), salinity—S(0) and chlorophyll *a* concentration—Ca(0) (mg m^−3^) near Glacier—G, Inner—I, and Outer—O zone of Kongsfjorden and Hornsund fjord within 1994–2019.

Zone	Pe(0)	T(0)	Sal(0)	Ca(0)
*Kongsfjorden*				
G				
Min	0.12	0.26	28.16	0.56
Max	6.24	6.7	31.35	0.6
Mean	2.18	3.23	29.65	0.58
SD	2.75	2.96	1.36	0.03
I				
Min	0.01	0.85	28.06	0.2
Max	8.14	6,7	31.69	1.14
Mean	2.2	3.76	30.4	0.68
SD	1.88	1.23	1.25	0.37
O				
Min	0.19	4.35	31.30	0,23
Max	2.74	6.62	34.96	1.23
Mean	1.53	5.69	33.48	0.67
SD	1.28	0.97	1.93	0.51
*Hornsund*				
G				
Min	0.83	3.51	29.79	0.80
Max	12.83	4.50	30.77	1.29
Mean	6.0	3.99	30.28	1.05
SD	5.39	0.54	0.52	0.34
I				
Min	0.38	1.7	29.78	0.27
Max	13.26	4.9	33	3.47
Mean	3.49	3.61	31.61	1.86
SD	4.21	1.13	0.87	1.51
O				
Min	1.24	1.75	28.5	0.75
Max	2.40	5.03	33.83	1.46
Mean	1.77	4.46	33.78	1.15
SD	0.51	0.64	0.06	0.36

Surface productivity in both studied fjords was higher in areas influenced by glacial waters, decreasing toward the fjords' outer parts. In Kongsfjorden, a gradual decline in the mean value of Pe(0) was observed, decreasing from 2.18 ± 2.75 mgC m^−3^ h^−1^ near the glacier to 1.53 ± 1.28 mgC m^−3^ h^−1^ in the outer part. In Hornsund, this decrease was from 6 ± 5.39 to 1.77 ± 0.51 mgC m^−3^ h^−1^. This pattern highlights a clear gradient in PP from the Glacier to the Outer zones of the fjords.

#### 3.2.3 Integrated primary production in the water column

**FIGURE 10:**
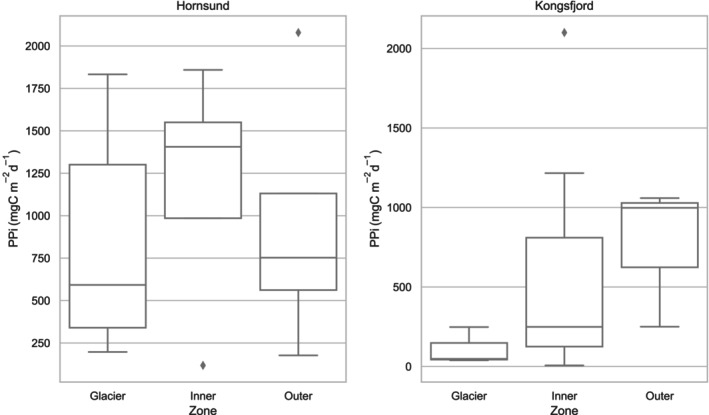
Range of integrated daily primary production Pi (mgC m^−2^ day^−1^) values in summer in Hornsund and Kongsfjord in studied regions within 1994–2019. The boxplot delineates the quartiles of the dataset, with the central box representing the interquartile range and the “whiskers” extending to depict the remainder of the data distribution, excluding points identified as outliers.

The results for the integrated value of primary production (Pi) in Hornsund reveal a considerably higher value than those in Kongsfjord (Figure 10). Pi values are 1.8 times higher in the Glacier zone of Hornsund compared to Kongsfjord (740.4 ± 746.4 mgC m^−2^ day^−1^ in Hornsund and 111.5 ± 118.0 mgC m^−2^ day^−1^ for Kongsfjord), and three times higher in the Inner zone of Hornsund (1203.0 ± 604.3 and 486.5 ± 562.2 mgC m^−2^ day^−1^, respectively). However, the difference in Pi values between the two fjords is less pronounced in the Outer zone, with Kongsfjord showing slightly higher average values (940.0 ± 807.69 and 769.12 ± 540.53 mgC m^−2^ day^−1^ for Hornsund and Kongsfjord, respectively).

**FIGURE 11:**
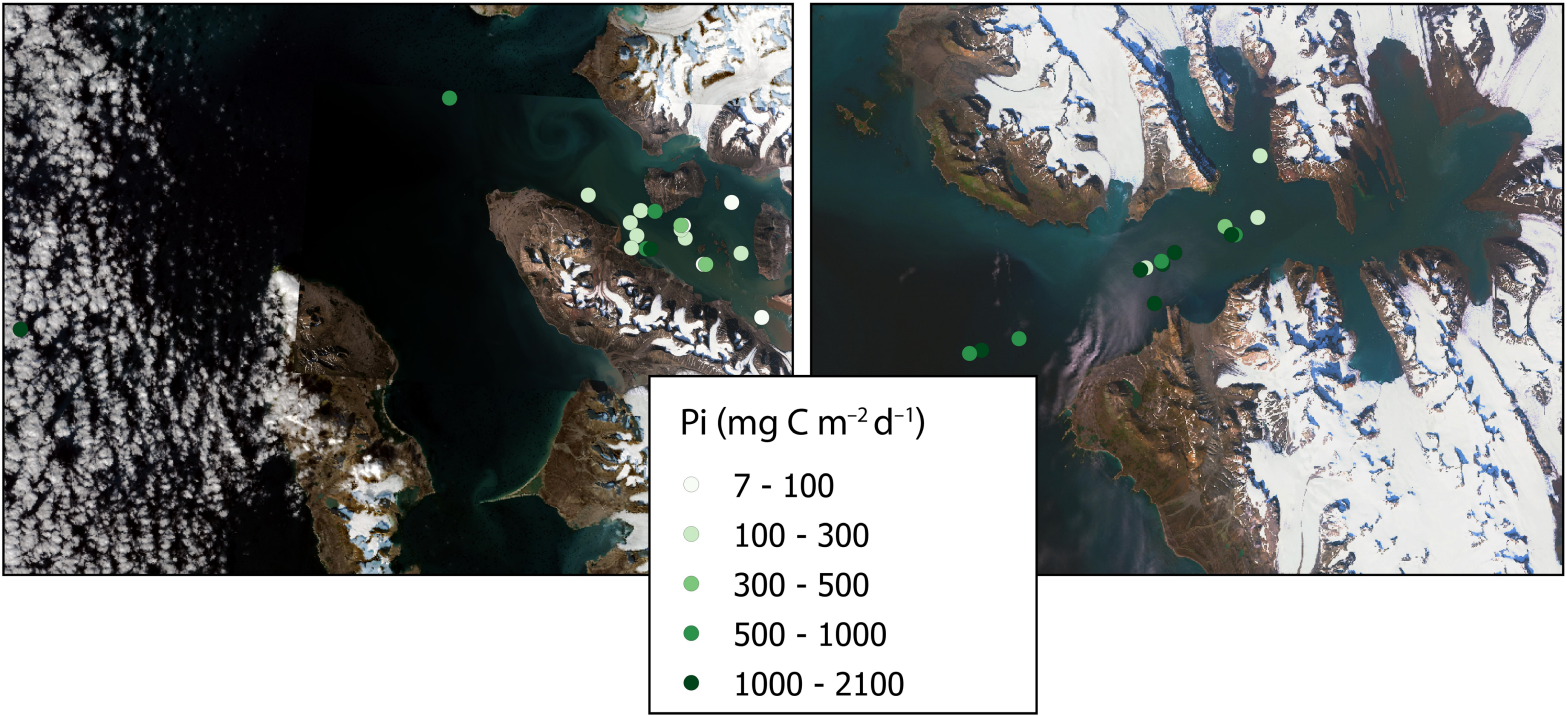
Spatial distribution of values of integrated primary production Pi (mgC m^−2^ day^−1^) in Kongsfjord (left figure) and Hornsund (right figure).

The spatial distribution of Pi suggests that the glacial regions of the fjords are the least productive (Figure 11). This is particularly interesting, especially when considering the highest surface value of Pe(0) recorded for these regions as mentioned in the previous chapter. The discrepancy may be explained by the high concentration of mineral particulate suspended matter near the glacier front, which hinders the penetration of light into deeper waters. The highest average Pi values in Kongsfjord were found in the Outer zone (769.12 ± 540.53 mgC m^−2^ day^−1^), while in Hornsund, the highest values were observed in the Inner zone (1203.02 ± 604.34 mgC m^−2^ day^−1^). Even though the statistical test results indicate that there are no statistically significant differences between the Glacier, Inner, and Outer zones in both studied fjords (*p* = .76 and *p* = .32 for Hornsund and Kongsfjord, respectively), clear patterns in productivity distribution were observed. These findings highlight the intricate relationship between glacial influence, light penetration, and primary productivity in these diverse fjord ecosystems.

## 4 DISCUSSION

The unique dataset of long‐term in situ data analyzed in this study allowed us to present a comprehensive description of Summer PP variability in the Spitsbergen region and its relation to spatial environmental changes.

Currently, an increasing amount of studies focused on the influence of glacier meltwaters on PP, especially regarding the Greenland Ice Sheet (Arrigo et al., 2017; Juul‐Pedersen et al., 2015; Lund‐Hansen et al., 2018; Murray et al., 2015). Only a few measurements and quantifications of PP have been published from the Spitsbergen fjord region (Eilertsen et al., 1989; Halbach et al., 2019; Hodal et al., 2012; Hop et al., 2006; Iversen & Seuthe, 2011; Piwosz et al., 2009; Smoła et al., 2017). However, none of these works compare the productivity in the areas influenced by tidewater glaciers and other regions over the analyzed period 1994–2019. Many investigations were conducted at only single locations, making direct comparisons between different fjord regions impossible. Therefore, in the presented investigation we explored three various fjord regions, in the same months, enabling us to make a thorough comparison of conditions in three different zones of the hydrological regime: Glacier, Inner, and Outer with very high‐depth resolution of the data. Our reflections presented in this article aim to address the gaps in our understanding concerning the impact of glaciers on marine productivity. The sampling spatial scale in our study was considerably smaller than in most of the similar studies in Greenland (a few hundred kilometers), where larger glaciers with higher discharge rates were observed, and as a consequence PP is affected on a scale of 30–80 km down‐fjord from the marine‐terminating glaciers (Calbet et al., 2011; Meire et al., 2017; Murray et al., 2015). The relationship between summer marine productivity and increased meltwater discharge around Greenland was found to be non‐linear, as demonstrated by Hopwood et al. (2018). Similarly, as in our study, the Pi values show two minima, one in the Glacier zones and the other in the Outer zones. While nutrient content may be highest close to the glacier, due to the ground being carved and washed away by the flowing glacier, light availability is limited to the surface layer. Conversely, at the fjord mouths, mineral particle loads are generally lower and thus light availability is increased, but there is a lack of nutrients in the AW during summer. The present study explores the small‐scale variability in the influence of glacier runoff on productivity. In both fjords, in the Glacier zone the highest mean value of PP was observed in the surface layer (Figures 8 and 9). However, the integrated value of PP in the water column was the lowest, as deeper layers were deprived of light access. This finding underscores the significance of examining such small‐scale variations to gain a more comprehensive understanding of the impact of glacier runoff on marine ecosystems. Even though the stations from different regions were located over a small distance (5 km), a horizontal hydrological gradient from the Glacier, Inner, and Outer waters was well‐pronounced.

**FIGURE 12:**
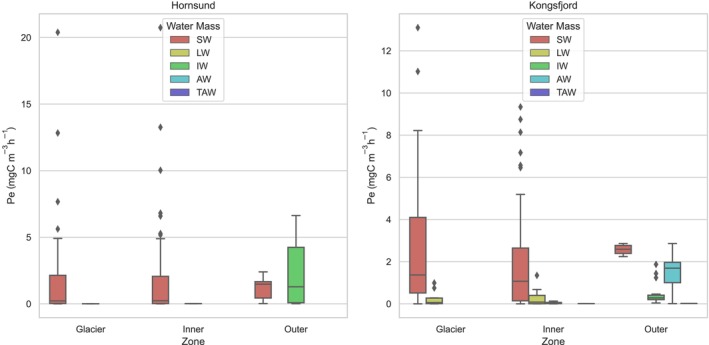
The bar graphs depict the value of primary production in various regions and different water masses. A bar plot represents a mean value and an error bar indicates the uncertainty around that estimate using standard error.

The results of this study are consistent with those of previous research, such as Hopwood et al. (2020), which suggests that marine‐terminating glaciers due to associated with the enhanced vertical fluxes of macronutrients typically lead to higher Pi in the surrounding areas, but on the other hand we can observe local suppression of PP due to light limitation. Field observations throughout the Arctic have unveiled considerable variations in PP across fjord ecosystems, with a notable influence from glacial inputs (Hop et al., 2002; Hopwood et al., 2020; Meire et al., 2017; Sejr et al., 2022; Stuart‐Lee et al., 2023). These studies indicate a broad spectrum of productivity levels, ranging from relatively low (<40 mgC m^−2^ day^−1^) to moderately high (>500 mgC m^−2^ day^−1^) during the meltwater season. Despite the statistical test results indicating no statistically significant differences between the Glacier, Inner, and Outer zones in both studied fjords, our research aligns with these observations, demonstrating big variability in the integrated value of primary production (Pi) across different zones and within our study area. Specifically, in the Hornsund fjord, Pi values in the Glacier zone were found to be 1.8 times higher than those in Kongsfjord, with the value of 740.4 ± 746.4 mgC m^−2^ day^−1^ in Hornsund compared to 111.5 ± 118.0 mgC m^−2^ day^−1^ in Kongsfjord. However, it is imperative to consider that the Glacier sampling stations in Hornsund were situated at a greater distance from the glacier front compared to the Glacier stations in Kongsfjorden. Notably, the latter were situated within the inner basin, separated from the outer basin by a submarine sill. The disparity was even more pronounced in Hornsund's Inner zone, where Pi values were three times greater than those in Kongsfjord (1203.0 ± 604.3 and 486.5 ± 562.2 mgC m^−2^ day^−1^, respectively). However, this difference was less evident in the Outer zone, where Kongsfjord exhibited slightly higher average Pi values. Contrasting these findings with the broader Arctic context, the pan‐Arctic basin, from March to September from 1998 to 2006, recorded a mean production rate of 420 ± 26 mgC m^−2^ day^−1^ (Pabi et al., 2008). Additionally, the Arctic shelf environments from May to August have shown a wide range of productivity, from 360 to 1500 mgC m^−2^ day^−1^ (Pabi et al., 2008), underscoring the dynamic and variable nature of PP in Arctic marine environments. These comparative insights emphasize the complex interplay between local glacial influences and broader climatic trends affecting PP in polar regions. It should be emphasized that in our research, despite the division research area into Glacier, Inner, and Outer zones, each of these zones, in varying degrees, is influenced by glacial waters, specifically SW (Figure 12). While the substantial levels of PP observed in the surface layer of the Glacier zone do not align with the vertical distribution of chlorophyll *a*, this discrepancy implies that the euphotic zone is significantly restricted in areas near glaciers. This limitation is largely attributed to the prevalence of mineral‐suspended matters, as highlighted by Dragańska‐Deja (2023). This condition may limit the depth to which light can penetrate, thereby affecting the spatial distribution of PP in regions influenced by glaciers (Sagan & Darecki, 2018). Despite the high concentration of chlorophyll *a* at a depth of 7 m, which indicates a substantial biomass of phytoplankton, the process of PP is hindered by insufficient light penetration. It is important to emphasize the crucial role of light availability in PP, especially in regions near glaciers where mineral suspensions can significantly reduce the depth of the euphotic zone.

At the front of the tidewater glacier in Greenland fjords, the concentrations of nutrients, specifically silicon (Si) and nitrate (NO_3_), exhibit an increase concomitant with a decrease in salinity. This pattern aligns with the characteristics of tidewater glacier runoff plumes, which are associated with the supply of nutrients, as corroborated by studies from Arimitsu et al., 2016, Fransson et al., 2017, Kanna et al., 2018, and Meire et al., 2016, 2023. In land‐terminating glacier fjords, where upwelling mechanisms are absent, nutrient dynamics show that dissolved Si concentrations increase at low salinities while nitrogen N levels remain consistently low. Studies in Kongsfjorden and other Arctic fjords have documented this pattern, with high silicic acid levels observed in glacially influenced waters, reflecting the terrestrial inputs from glacial meltwater. Meanwhile, nitrogen levels are typically low due to limited biological uptake and regeneration processes in these cold environments (Hopwood et al., 2020; Meire et al., 2017). Recent observations in Kongsfjorden have also indicated that chlorophyll *a* concentrations tend to be higher in the inner parts of the fjord compared to the Outer parts, which is consistent with our findings. The distribution of autotrophic protists in examined fjords also exhibited dependence on environmental gradients (Assmy et al., 2023; Piwosz et al., 2009; Wiktor & Wojciechowska, 2005). Tracking the results of qualitative‐quantitative research on protist composition and their spatial distribution carried out during voyages, in which PP measurements were mostly conducted, it can be inferred that the closer to the glaciers, the fewer autotrophic diatoms occurred, being replaced by mixo‐ or heterotrophic nanoflagellates of undefined taxonomic affiliation and dinoflagellates.

Furthermore, it is important to note that the variability in PP values considered over the research period is influenced by fjord‐specific conditions. These conditions include factors such as the interplay of the inflow of the Atlantic and runoff water, ambient air temperature, tidal current, and wave dynamics. Such environmental factors play a pivotal role in shaping the local PP dynamics within fjord ecosystems, underscoring the complexity and variability in these marine environments. Variability in the value of PP observed between Kongsfjord and Hornsund can be explained by the different geometry of the fjords, with Hornsund featuring a more intricate coastline that includes several bays (e.g., Burgerbukta and Samarinvågen) absent in Kongsfjorden. Additionally, the distinct dynamics of water exchange with the shelf and water column in both fjords play a significant role. Kongsfjorden is under the strong influence of relatively warm Atlantic‐derived water, while Hornsund experiences temporary inflows of the cold Arctic water transported from Sorkapp Current (Promińska et al., 2017). In the light of ongoing warming, for example, in the years 2001–2015, the Kongsfjord was affected more often by AW resulting in a mean value of temperature of 2.9°C and salinity of 34.5, while in Hornsund—influenced by the cold Sørkapp Current—mean water temperature was equal 2.12°C and salinity 34.06 (Promińska et al., 2017). The water in Kongsfjorden is, on average, warmer by approximately 1°C and exhibits a salinity higher by 0.5 compared to Hornsund. This fjord is also distinguished by a twofold greater influx of AW and associated heat delivery. Both fjords are experiencing a progressive warming trend, primarily due to the increased incursion of waters of Atlantic origin. During the summer months, the air temperature in Kongsfjorden is generally higher than that of Hornsund (Cisek et al., 2017).

Despite these changes, Hornsund continues to maintain a more Arctic‐type fjord characteristic compared to Kongsfjorden. This differential impact of ocean currents on the two fjords underscores the complex interplay of regional oceanographic processes affecting biological productivity, species composition, and overall ecological conditions. These differences also affect the availability of the nutrients that constrain local PP (Arimitsu et al., 2016; Calleja et al., 2017; Hopwood et al., 2020; Meire et al., 2017; Skrzypek et al., 2015). It should be noted that Arctic Ocean ecosystems are characterized by strong spring phytoplankton blooms that typically move with the receding ice within the seasonal ice zone (Carmack & Wassmann, 2006; Sakshaug, 2004). However, it should be emphasized that our measurements were conducted during the summer period, which means that these observations did not capture the maximum PP values that typically occur during the spring.

The regions proximate to the glaciers, where high values of surface PP have been recorded, act as a kind of trap for plankton, causing its demise (Gluchowska et al., 2016; Zajaczkowski & Legezyńska, 2001). This leads to a high delivery of organic matter to the seabed layers (Bourgeois et al., 2016; Herbert et al., 2021), which corresponds with areas of krill aggregation at the seabed, as identified by Deja et al. (2019). It is hypothesized that the krill are likely feeding on the sedimenting phytoplankton in these areas. These regions also serve as a “cafeteria” for seabirds (Stempniewicz et al., 2017). The circulation patterns and gradients in salinity and turbidity resulting from glacier meltwater discharge can lead to the stunned and entrapment of zooplankton, thereby attracting top predators (Hop et al., 2002; Urbanski et al., 2017; Węsławski et al., 2000). This new and complex primary research provides new insights into the underlying reasons for the formation of seabird hotspots, as documented by Dragańska‐Deja et al. (2020) and Urbanski et al. (2017), as well as the presence of marine mammals, as reported by Lydersen et al. (2014). These findings suggest a significant ecological link where areas of high PP, indicative of rich phytoplankton growth, serve as feeding grounds for krill, which in turn attract higher trophic levels such as seabirds and marine mammals. This chain of interactions highlights the intricate web of marine ecosystem dynamics, particularly in glacial‐influenced regions.

### 4.1 Satellite point of view

Nowadays, PP is most commonly estimated from remote sensing measurements based on the sea surface temperature, PAR, and chlorophyll *a* concentration (Cherkasheva et al., 2023; Lee et al., 2015, 2016). The utilization of remote sensing algorithms for estimating PP in oceanic waters, where the optical properties are primarily influenced by organic suspended matter, has proven to be a reliable method for determining PP values with a reasonable degree of accuracy. Nevertheless, studies have shown that in the open waters of the Arctic, models tend to more accurately replicate primary productivity using in situ chlorophyll *a* measurements rather than satellite‐derived values. This finding highlights the need for careful calibration and refinement of Net primary productivity models to suit the specific conditions of the Arctic Ocean (Lee et al., 2015). However, the application of these remote sensing algorithms to fjord ecosystems encounters numerous challenges. Firstly, these waters contain a large amount of suspensions of terrestrial origin, in which the absorption properties are influenced not only by organic suspensions—phytoplankton but also by inorganic—mineral ones with various absorbing and dispersing characteristics and dissolved substances (Pegau, 2002; Sagan & Darecki, 2018). Secondly, it is an environment of clashing and mutual interactions of two types of water—Arctic and those coming from melting glaciers, the intensity of which varies in subsequent years (Błaszczyk et al., 2009, 2013; Cottier et al., 2010). Additionally, the sea surface in these regions is often obscured by cloud cover. The presence of sunlight, sea ice, or icebergs further alters the optical signals originating from the surface layers, complicating remote sensing efforts. Furthermore, it is important to note that the maxima of chlorophyll *a* or PP typically occurs in deeper water layers as presented in this study, beneath the depth that can be effectively monitored by remote sensing methods. As a result, detecting these sub‐surface maxima via satellite observations is generally not feasible. This highlights the complexities and limitations inherent in the remote sensing of aquatic environments, especially in those influenced by glaciers.

**FIGURE 13:**
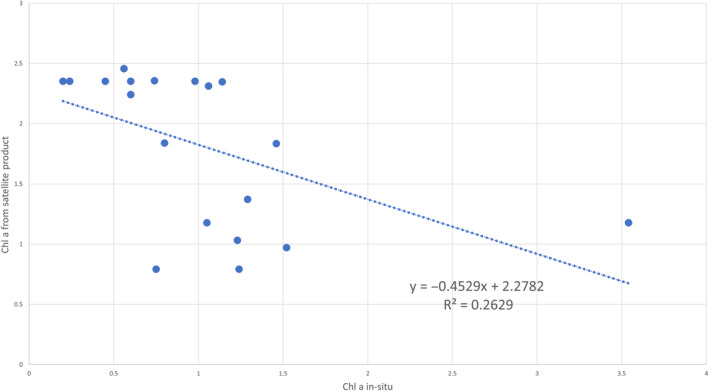
Scatter plot comparing in situ measurements of chlorophyll a (in situ Ca) concentrations against those derived from the MODIS satellite product.

**FIGURE 14:**
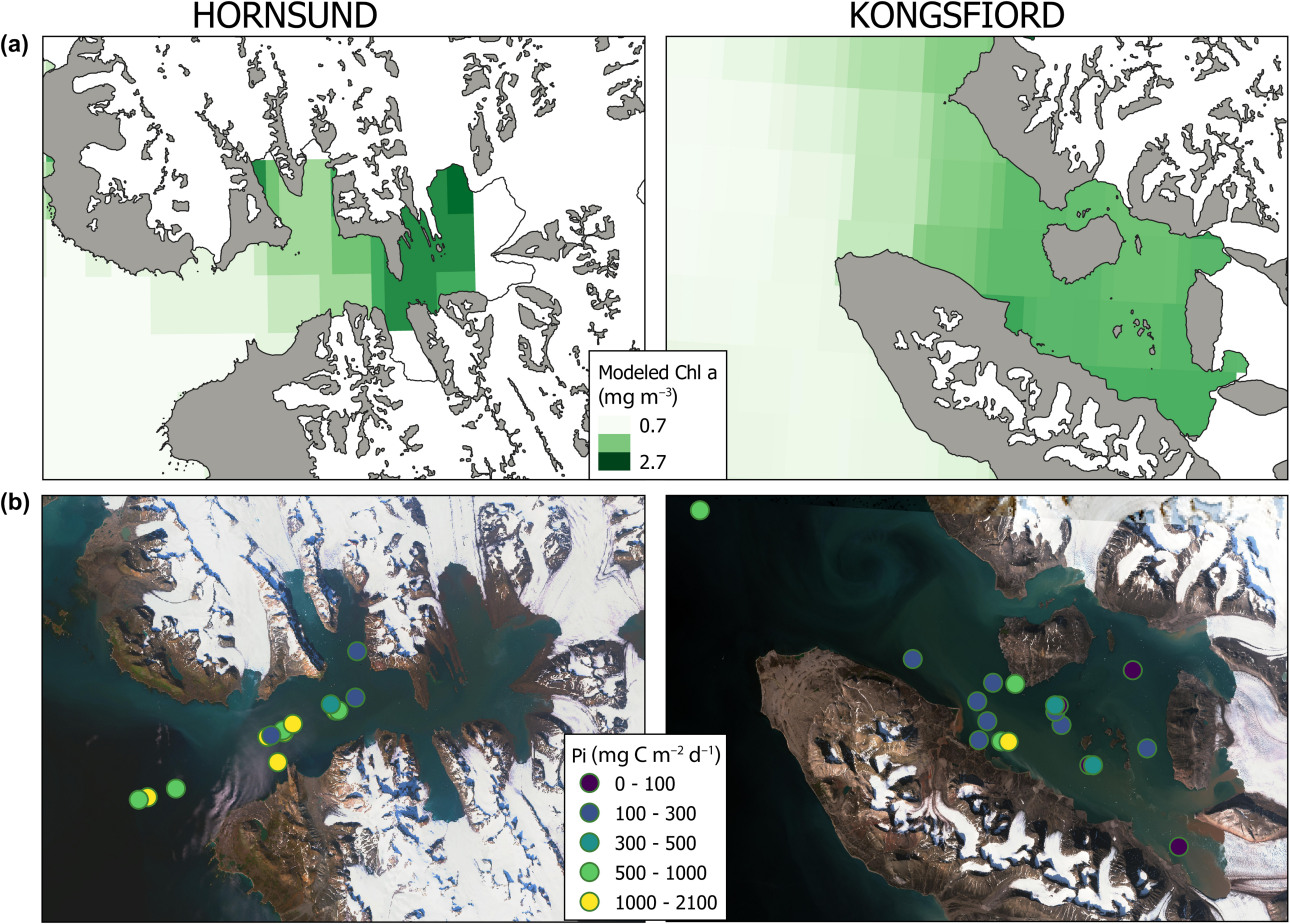
Panel (a) displays the average values of chlorophyll *a* concentrations for Hornsund and Kongsfjord derived from the MODIS AQUA product, aggregating data collected during July over the years 2002–2022. Panel (b) presents a true color RGB composition obtained from Sentinel 2, in 2019.07.27 for Hornsund and 2019.07.28 for Kongsfjord, illustrating glacier meltwater runoff—referred to as the brown zone. Points represent the Pi value at the sampling sites.

Chlorophyll *a* algorithms that utilize satellite data tend to overestimate chlorophyll content, primarily due to the presence of colored dissolved organic matter (CDOM) in the water, mineral suspensions, and the influences of land and icebergs, which can affect the satellite signal. Recent studies indicate that in Svalbard fjords, there is an observed increase in the seasonal release of glacial runoff, accompanied by a positive trend in the darkening of seawater in this region (Konik et al., 2021; Szeligowska et al., 2022). The correlation between runoff and suspended sediment load is complex, with significant variability observed at the catchment level. Increased runoff generally results in higher suspended sediment loads, although this relationship can be influenced by factors such as glacial dynamics and precipitation patterns (Chu et al., 2009). Consequently, caution is advised when drawing conclusions based solely on chlorophyll estimates generated by global algorithms. Figure 13 illustrates this discrepancy, showing that the average chlorophyll values for a specific point in July, as estimated by satellite data, can be up to three times higher than those measured in situ. This relationship is inversely proportional, meaning that high values of suspended solids and CDOM in glacial regions are often misinterpreted as high chlorophyll *a* concentrations by satellite algorithms. As a result, the chlorophyll‐a values derived from these algorithms are overestimated and do not align with in situ measurements. This discrepancy is starkly evident in Figure 14, where a satellite image in natural colors (RGB composite) depicts the flow of suspended matter, also referred to as the “brown zone.” Contrasting this natural color imagery, the maps showing chlorophyll *a* values calculated based on satellite data indicate abnormally high values in these same regions. This divergence underscores the challenges in accurately assessing chlorophyll concentrations in glacial and fjord waters using standard remote sensing algorithms and highlights the importance of corroborating satellite data with direct in situ measurements.

The resolution to the challenge of accurately monitoring fjord environments lies in the availability of in situ data and the development of local algorithms tailored for high‐resolution satellite data. Fjords, with their dynamic and variable nature, present a unique challenge wherein satellite algorithms, typically effective in oceanic waters, may incur significant errors. Hence, the analyses presented in this work, grounded in data from in situ measurements, are of exceptional value. They provide insights into the changes occurring within the fjord environment. Building upon this foundation, it becomes possible to correlate, modify, and verify satellite algorithms that aim to depict environmental and biogeochemical parameters on a broader spatial and temporal scale.

### 4.2 Conclusions

The extensive analysis of PP within the Spitsbergen fjords, specifically Kongsfjorden and Hornsund, over the period 1994–2019, has yielded significant insights into the dynamic changes occurring in these polar ecosystems. This study, integrating data from 45 measurement experiments, has significantly contributed to our understanding of how melting glacier waters and AW influence the production of organic matter in these regions. The observed low values of integrated PP in Glacier regions can be attributed to the constraining effect of meltwater on the depth of the euphotic zone. The research revealed marked spatial variations in PP across different zones of the fjords. The highest values were observed in the surface layers of the Glacier and Inner zones, particularly in Hornsund, with a notable decline in productivity at increased depths. Notably, surface PP [Pe(0)] in Hornsund's Glacier zone was found to be 2.8 times greater and 1.6 times greater in the Inner zone than the corresponding average values in Kongsfjord. The disparity was least pronounced in the Outer zones of the fjords, where Hornsund demonstrated only a 1.2‐fold increase compared to Kongsfjord. Furthermore, Pi values in Hornsund significantly exceeded those in Kongsfjord, being 1.8 times higher in the Glacier zone and threefold higher in the Inner zone. However, this distinction in Pi values became less marked in the Outer zone, with Kongsfjord presenting slightly elevated average values. This finding underscores the complex interplay between environmental factors and PP in Arctic marine ecosystems. While satellite observations offer extensive coverage and valuable insights, this research emphasized the necessity of in situ measurements for validation and calibration, particularly in regions with dynamic environmental changes like Spitsbergen fjords. The findings from this study pave the way for future research, especially in the context of ongoing climate change. Continued monitoring and analysis, combining both satellite and in situ data, will be vital in enhancing our understanding of the complex mechanisms governing PP and overall ecosystem health in dynamic polar fjords.

In summary, this study not only enhances our understanding of PP dynamics in the Spitsbergen fjords elucidating the effects of environmental factors such as light availability, depth, and glacier influence on marine productivity in these Arctic ecosystems but also highlights the importance of integrating diverse research methodologies to assess the impact of environmental changes in polar ecosystems.

## AUTHOR CONTRIBUTIONS

**Katarzyna Dragańska‐Deja:** Conceptualization (equal); data curation (equal); formal analysis (equal); investigation (equal); methodology (equal); validation (lead); visualization (lead); writing – original draft (equal); writing – review and editing (equal). **Joanna Stoń‐Egiert:** Conceptualization (equal); data curation (equal); formal analysis (equal); investigation (equal); methodology (equal); writing – original draft (equal); writing – review and editing (equal). **Józef Wiktor:** Writing – review and editing (supporting). **Mirosława Ostrowska:** Conceptualization (supporting); writing – review and editing (supporting).

## CONFLICT OF INTEREST STATEMENT

The authors declare that they have no conflict of interest.

## Data Availability

The data are available at https://doi.org/10.48457/iopan‐2024‐198.
